# Remote and reversible inhibition of neurons and circuits by small molecule induced potassium channel stabilization

**DOI:** 10.1038/srep19293

**Published:** 2016-01-13

**Authors:** Eva Auffenberg, Angela Jurik, Corinna Mattusch, Rainer Stoffel, Andreas Genewsky, Christian Namendorf, Roland M. Schmid, Gerhard Rammes, Martin Biel, Manfred Uhr, Sven Moosmang, Stylianos Michalakis, Carsten T. Wotjak, Christoph K. Thoeringer

**Affiliations:** 1 Department of Internal Medicine II, Klinikum rechts der Isar, Technical University of Munich, Germany; 2 Institute of Anesthesiology, Technical University of Munich, Germany; 3 Max Planck Institute of Psychiatry, Department of Stress Physiology and Neurogenetics, Munich, Germany; 4 Center for Integrated Protein Science Munich (CIPSM) and Department of Pharmacy – Center for Drug Research, Ludwig-Maximilians-University of Munich, Germany; 5 Institute of Pharmacology, Technical University of Munich, Germany

## Abstract

Manipulating the function of neurons and circuits that translate electrical and chemical signals into behavior represents a major challenges in neuroscience. In addition to optogenetic methods using light-activatable channels, pharmacogenetic methods with ligand induced modulation of cell signaling and excitability have been developed. However, they are largely based on ectopic expression of exogenous or chimera proteins. Now, we describe the remote and reversible expression of a Kir2.1 type potassium channel using the chemogenetic technique of small molecule induced protein stabilization. Based on shield1-mediated shedding of a destabilizing domain fused to a protein of interest and inhibition of protein degradation, this principle has been adopted for biomedicine, but not in neuroscience so far. Here, we apply this chemogenetic approach in brain research for the first time in order to control a potassium channel in a remote and reversible manner. We could show that shield1-mediated ectopic Kir2.1 stabilization induces neuronal silencing *in vitro* and *in vivo* in the mouse brain. We also validated this novel pharmacogenetic method in different neurobehavioral paradigms.The DD-Kir2.1 may complement the existing portfolio of pharmaco- and optogenetic techniques for specific neuron manipulation, but it may also provide an example for future applications of this principle in neuroscience research.

Studying how chemical and electrical signals affect the function of neurons and circuits that translate these signals into behavior remains one of the major challenges in neuroscience. In recent years, several neurogenetic approaches have been developed to manipulate neuronal activity in the brain of living animals for studying its effects on behaviour and synaptic plasticity like optogenetic tools^1^ or small molecules and designer receptors^2^,^3^ with significant impact on neuroscience. However, most of these neurotechniques rely on the ectopic expression of exogenous or chimera proteins, or require activation by non-physiological stimuli. Alternatively, controlling endogenous proteins involved in neuron and circuit modulation has been reported using photo-inducible potassium channels^4^. This elegant tool, however, lacks reversibility that constrains its potential use in neuroscience.

Therefore, we searched for an alternative strategy to exert selective, remote and reversible inhibitory control on neurons and circuits in living animals by controlling proteins intrinsically regulating the membrane potential. We adapted a pharmacogenetic method for direct pharmacological control of protein stability^5^. This genetic tool utilizes a mutant destabilizing domain (DD) of human FKBP12 protein that can be attached to virtually any protein of interest. Within the cell, the system harness cellular protein degradation systems to reduce newly synthesized DD proteins. A synthetic, cell permeant, biologically otherwise inert ligand (shield1)^5^ binds to the FKBP12-derived destabilizing domains and shields DD proteins from degradation, allowing fused proteins to perform their cellular functions. The shield1 - DD tool has already been proven efficient for *in vivo* applications in cancer animal models^6^ and for stabilization of the Cre recombinase, however, by a different stabilization system^7^.

In the present study, we probed the small molecule DD tool for modulating brain function and fused DD of human FKBP12 to Kir2.1 at the N-terminus, an inwardly rectifying potassium channel. It primarily regulates neuronal excitability and action potential cessation by membrane hyperpolarization^8^,^9^ and the ectopic expression of these channels has been used in the past to study neuronal networks^10^,^11^. Here, we hypothesized that shield1-induced ectopic DD-Kir2.1 stabilization/overexpression inhibits neuronal firing by hyperpolarization and, finally, modulates specific behaviors of an animal. In the absence of shield1, however, DD-Kir2.1 is degraded by the proteasome leaving neuronal function unaffected.

## Results

### Inhibitory effects of shield1-induced Kir2.1 stabilization *in vitro* and *in vivo*

**Figure 1:**
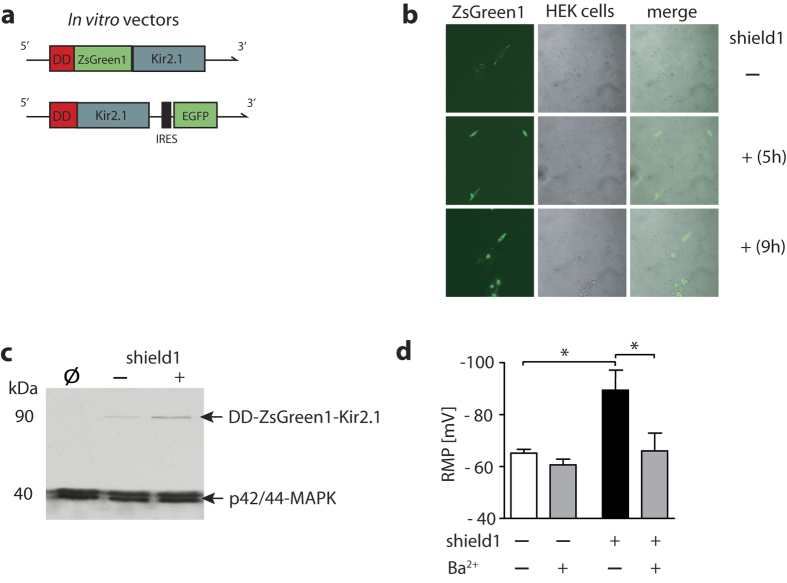
Neurophysiological effects of shield1-dependent Kir2.1 stabilization *in vitro*. (**a**) Schematic representation of the DD-Kir2.1 genetic constructs used for *in vitro* experiments with HEK293T cells. *Top*: DD-ZsGreen1-Kir2.1 for experiments in (**b,c**). *Bottom*: DD-Kir2.1-IRES-GFP for experiments in (**d**). DD, destabilizing domain. GFP, green fluorescent protein. IRES, internal ribosomal entry site. Kir2.1, Kir2.1 inward-rectifier potassium ion channel. ZsGreen1, Zoanthus green fluorescent protein. (**b**) Images presenting the pattern of ZsGreen1 expression at different time points after shield1 incubation (500 nM) vs. baseline (no ligand present [Ø]). (**c**) Treating HEK cells with shield1 (500 nM; 4 h) also increased the amount of the DD tag compared to ligand-free (shield1 -) and non-transfection conditions (Ø). (**d**) Resting membrane potentials (RMP) were measured by patch-clamp recordings in HEK293T cells transfected with the DD-Kir2.1-IRES-EGFP vector and treated with ( + )/without (−) shield1 (500 nM; 3–4 h incubation). Ligand-induced hyperpolarization was reversed by extracellular Ba^2 + ^(300 μM). *p < 0.05 (Student’s t-test), ^#^p < 0.05 (Newman-Keuls posthoc test), data are plotted as mean ± S.E.M.; number of cells in (**d**) n = 7 (shield1 −), 8 (shield1 + ).

In a first proof-of-principle *in vitro* experiment, we expressed DD-Kir2.1 via a pcDNA3.1 plasmid in HEK293T cells (Fig. 1a; DD-ZsGreen1-Kir2.1). Addition of shield1 to the cell culture stabilized DD-ZsGreen1-Kir2.1 expression in HEK cells in a time-dependent manner as ZsGreen1 fluorescence increased after drug incubation for 5 h (Fig. 1b). Likewise, immunoblotting revealed an increased amount of the DD fusion protein 4 h after adding the ligand compared to the non-shield1 or non-transfection conditions (Fig. 1c). A faint protein band indicates minimal expression of the fusion protein in HEK293T cells under basal conditions (i.e., transfection, but no shield1 treatment). The ratios of p42/44-MAPK/DD-Kir2.1 grey values were [mean ± S.E.M; 4 replicates]: Ø = 0.59 ± 0.09; DD-Kir2.1 without shield1 = 0.62 ± 0.08; DD-Kir2.1 with shield1 = 0.74 ± 0.10. Next, after constructing an additional vector for *in vitro* experiments, we recorded the resting membrane potential (RMP) in DD-Kir2.1-pIRES-GFP vector-transfected HEK293T cells and found a shield1-induced shift towards a significantly more negative RMP (Δ RMP = −20 mV) that could be reversed by adding barium chloride (Ba^2+^), a selective Kir blocker, to the bath solution (Fig. 1d).

**Figure 2:**
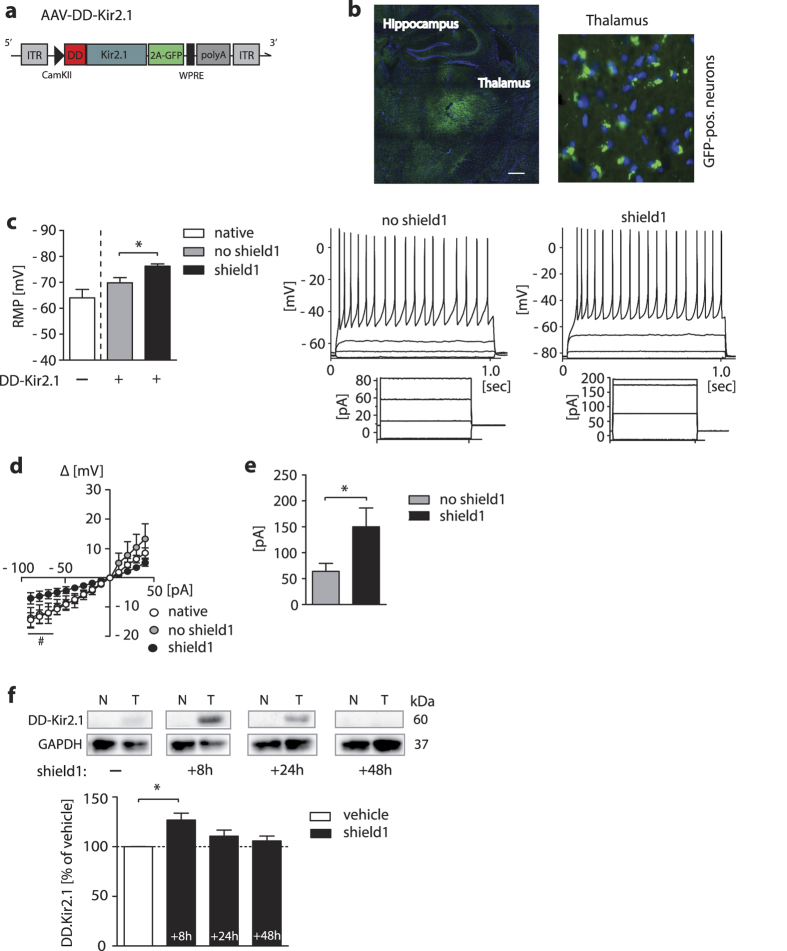
Effects of shield1-dependent Kir2.1 stabilization on neuron characteristics *in vivo*. (**a**) Schematic representation of the viral construct used to transduce neurons with DD-Kir2.1, AAV2.1-CamKII-DD-Kir2.1-2A-GFP-WPRE; CamKII, Ca^2 + ^/calmodulin-dependent protein kinase II promoter. WPRE, Woodchuck hepatitis posttranscriptional regulatory element. ITR, inverted terminal repeat. polyA, human growth hormone poly A-addition signal. (**b**) *Left*: Representative image of virus-induced GFP-fluorescence revealing the extend of virus infection of the left dorsolateral thalamus; area used for electrophysiogical patch-clamp experiments in acute mouse brain slices (scale bar, 400 μm). *Right*: Cytosolic GFP fluorescent thalamic neurons (40× magnification). DAPI was used to counterstain nuclei. (**c**) *Left*: RMP analysis of thalamic neurons (native [no virus injection] vs. AAV-DD-Kir2.1 transduced [DD-Kir2.1 + ; GFP + ]) treated with or without shield1 (500 nM; 2–4 h incubation). *Right*: Representative whole-cell current-clamp recordings of AP series in thalamic neurons (AAV DD-Kir2.1 + ; no shield1 vs. shield1 treatment) with voltage traces plotted upon current injections; steps of the current-clamp protocol are shown below. (**d**) I–V relationsship in native neurons vs. neurons transduced with AAV-DD-Kir2.1 and w/o shield1 treatment (500 nM; 2–4 h incubation) obtained by current injections (10 pA increments) and recording of the resulting voltage (mV) over the membrane. (**e**) Presentation of the rheobase as minimum current required to fire an action potential in DD-Kir2.1 transduced neurons incubed with shield1 (500 nM; 2–4 h incubation) vs. controls. (**f**) Immunoblotting analysis of striata from saline- and shield1-injected mice isolated at different time points after injection and probed for DD and GAPDH (loading marker); N = striatum, no virus; T = contralateral striatum, side with AAV-DD-Kir2.1 delivery. *p < 0.05 (Student’s t-test), ^#^p < 0.05 (Newman-Keuls posthoc test), data are plotted as mean ± S.E.M.; number of cells in (**c**–**e**) n = 10 (native), 12 (shield1 −), 16 (shield1 + ); number of mice in (**f**) n = 3 (vehicle), 3 (shield1 per time point).

To probe our approach *in vivo*, we used an AAV8YF-pseudotyped adeno-associated virus (AAV) vector and expressed DD-Kir2.1-2A-GFP under control of a Ca^2+^/calmodulin-dependent protein kinase alpha (CamKIIα)-promoter^12^ (AAV2.1-CamKII-DD-Kir2.1-2A-GFP, referred to *AAV-DD-Kir2.1* for the rest of the manuscript) for expression of the fusion protein in neurons of living mice (Fig. 2a). For intracellular electrophysiological recordings, AAV-DD-Kir2.1 was stereotactically injected in the dorsolateral thalamus of C57BL/6 (B6) mice (Fig. 2b), a brain area with predominant excitatory pyramidal neurons, and acute brain slices were prepared after a transduction period of 3-4 weeks. As observed in HEK293 cells, shield1-induced stabilization of ectopic Kir2.1 significantly hyperpolarized the RMP in transduced neurons compared to transduced neurons without ligand treatment (ANOVA: F_2,37_ = 15.0; p < 0.0001; Δ RMP = −9 mV), without a significant difference between native (no virus injection) and transduced-only cells (Fig. 2c). As an additional parameter of passive membrane properties, which is potentially influenced by our tool, we calculated *R*_in_ and found significant treatment differences (native: 170.1 ± 25.0 MΩ, no shield1: 172.4 ± 34.4 MΩ, shield1: 78.6 ± 21.7 MΩ [mean ± S.E.M.], ANOVA: F_2,26_ = 4.5; p = 0.02). A current-voltage relationship is presented in Fig. 2d reflecting the decrease in input resistance by drug-induced DD-Kir2.1 overexpression as presented previously^11^. To assess the excitability of cells with shield1-mediated DD-Kir2.1 stabilization, we calculated the rheobase and observed that the current needed to evoke minimal action potential firing in shield1-treated neurons was significantly higher than in non-treated cells (Fig. 2e) confirming the predicted decrease in excitability of neurons. Of note, incubating neuronal cells with the ligand shield1 (500nM; 2–3 h) itself did not influence any of the electrophysiological properties in native neurons (RMP [mean ± S.E.M.]: native: 65.5 ± 3.983 mV, shield1: 62.0 ± 4.83 mV [t-test: p = 0.72]; *R*_in_ [mean ± S.E.M.]: native: 178.4 ± 35.9 MΩ, shield1: 208.5 ± 52.2 MΩ [t-test: p = 0.53]; rheobase [mean ± S.E.M.]: native: 78.5 ± 9.4 pA, shield1: 92.5 ± 12.5 pA [t-test: p = 0.32]).

We also injected AAV-DD-Kir2.1 stereotactically into the striatum of C57BL/6 (B6) mice to assess the time dependent effects of shield1-induced Kir2.1 stabilization. Immunoblotting revealed that DD expression was significantly upregulated in the virally transduced site of the striatum only 8h after drug injection and faded at the later time points. No DD was detected in the contralateral site of the striatum (Fig. 2f).

### Small molecule-induced neuron inhibition in a pharmacological challenge test in mice

In a series of experiments, we evaluated the applicability of shield1-induced DD-Kir2.1 stabilization and neuronal silencing for studying behavioral processes in mice.

As there are no data available concerning the pharmakokinetic characteristics of shield1, we performed an additional set of experiments, in which we assessed blood and brain levels of the ligand at different time points after administration. Mice received a single i.p. injection of shield1, and tissue levels were measured 8 h, 24 h and 48 h afterwards by mass spectrometry. Shield1 could be detected in the mouse brain and serum 8h after a single application (brain [mean ± S.E.M]: 2.2 ± 0.2 ng/g wet tissue; serum [mean ± S.E.M]: 28.0 ± 4.1 ng/ml), but it was below detection limit at later time points (brain < 1.5 ng/g wet tissue, serum < 10 ng/ml; 3 mice per group). These kinetic data support the behavioral and immunoblotting results, where small-molecule modulation occurs only at early time points.

**Figure 3:**
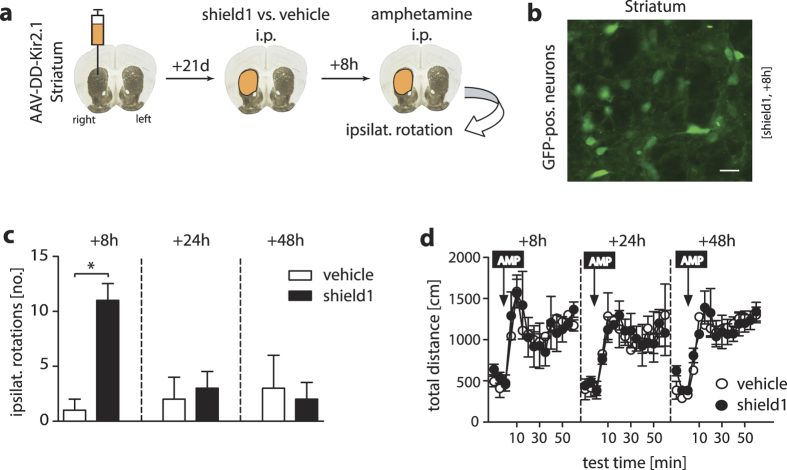
Behavioral characteristics of shield1-induced Kir2.1 stabilization and related neuron inhibition *in vivo* in a pharmacological stimulation test. (**a**) Schematic of the experimental design of the amphetamine-induced stereotype rotation behavior. Unilateral virus injection into the mouse striatum (40×). (**b**) Typical image of GFP-positive striatal neurons transduced with AAV-DD-Kir2.1 after shield1 administration (scale bar, 20 μm). (**c**) Analysis of amphetamine (AMP)-induced (2.5 mg/kg; i.p.) ipsilateral rotations in an open field (OF) arena in mice that received unilateral striatum injections of AAV-DD-Kir2.1, and were i.p.-injected once with shield1 (10 mg/kg) or vehicle (saline). Shield1-treated mice displayed significantly more stereotype rotations 8 h after treatment, but rotations returned to control values after 24 h. (**d**) Basal locomotion (total distance moved) and AMP-triggered rise in horizontal locomotor activity were not affected by ligand treatment. *p < 0.05 (Student’s t-test); data are plotted as mean ± S.E.M.; number of mice in (**c,d**) n = 4 (vehicle), 4 (shield1).

On the behavioral level, we chose a simple paradigm with a robust and reproducible phenotypic response that is sensitive to inhibition of neuronal activity. The amphetamine-induced stereotype rotation test has been frequently used to investigate disruptions in neuron function of the striatum in animal models of Parkinson’s disease, but also for validation of chemogenetic tools with neuronal silencing functions^2^. The principle is as follows: Upon unilateral damage or inhibition of the striatum, amphetamine (AMP) stimulates dopamine neurotransmission in the contralateral striatum causing rotations towards the lesioned, ipsilateral side (Fig. 3a)^13^. Here, we injected our AAV-DD-Kir2.1 virus into the right striatum of B6 mice (Fig. 3b). After recovery, we administered vehicle or shield1 systemically (10 mg/kg, i.p.; dose according to ref. 6) to trigger DD-Kir2.1 expression in striatal neurons. After an 8 h-time interval, a period necessary for shield1 to exert its effects on protein stabilization *in vivo*^6^, we administered AMP (2.5 mg/kg; i.p.) in every animal. As presented in Fig. 3c, the drug significantly increased the total number of ipsilateral rotations triggered by AMP. 24 h and 48 h after the ligand injection, rotations were no longer provoked by AMP indicating a reversible and transient inhibitory control on neuronal striatal function (Fig. 3c). AMP itself induced hyperlocomotion during each testing time point ruling out pharmacological desensitization after repeated AMP administration (Fig. 3d).

### Selective pharmacogenetic modulation of context-dependent fear memory in animals

**Figure 4:**
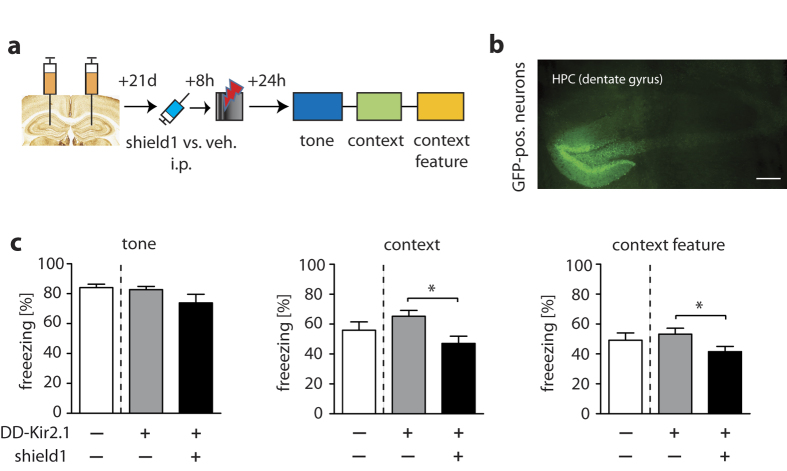
Shield1-induced neuronal inhibition and aquisition of fear memories *in vivo*. (**a**) Schematic representation of the fear conditioning experiment probing the effects of shield1-DD-Kir2.1 in the acquisition of fear memory. Mice received bilateral AAV-DD-Kir2.1 injections in the dendate gyrus of the dorsal hippocampus (HPC). 8 h before fear conditioning (a single pairing of a 20 sec-tone with a 2 sec-foot shock [1.5 mA]) mice were treated with shield1 (10 mg/kg; i.p.) or vehicle (saline; i.p.). (**b**) Typical mage of GFP fluorescence in the dendate gyrus after virus injection (scale bar, 200 μm). (**c**) *Left*: Freezing in percent of total test time to a conditioned tone in a neutral test context in control animals (no shield1/vehicle, no AAV transduction [DD-Kir2.1 −/Shield1 −]) vs. DD-Kir2.1 carriers (no shield1/vehicle, but AAV transduction [DD-Kir2.1 + /Shield1 −]) and shield1-injected mice ([DD-Kir2.1 + /Shield1 + ]) 24 h after the conditioning procedure. *Middle*: Significantly reduced freezing in the initial shock context (24 h after conditioning; no shock reinforcement) in the shield1-treated group compared to vehicle-injected DD-Kir2.1 carrier mice. *Right:* Significantly reduced freezing in a different context containing only a context feature (grid floor; 24 h after conditioning; no shock reinforcement) in the shield1-treated group compared to vehicle-injected DD-Kir2.1 carrier mice. *p < 0.05 (Student’s t-test), data are plotted as mean ± S.E.M.; number of mice in (**c**) n = 8 (DD-Kir2.1 −/Shield1 −), 10 (DD-Kir2.1 + /Shield1 −), 10 (DD-Kir2.1 + /Shield1 + ).

In addition to the pharmacological challenge test, we also probed DD-Kir2.1 physiologically in a mouse fear memory paradigm. We subjected mice to fear conditioning with combined auditory and contextual cues (Fig. 4a), with the latter being sensitive to perturbations in hippocampal circuits^14^. We injected AAV-DD-Kir2.1 bilaterally into the mouse hippocampus and observed vector expression mainly in the dentate gyrus (DG) (Fig. 4b). We then administered vehicle or shield1 (10 mg/kg) systemically (i.p.) 8 h before tone-foot shock conditioning to evaluate the efficacy of ligand-DD-Kir2.1-driven neuron silencing on fear memory acquisition. As expected, auditory fear memory was not affected by the drug as assessed by freezing to the conditioned tone 24 h after the conditioning session (Fig. 4c, *left*). However, freezing in the original shock context or in a different context containing only a feature of the shock context (i.e., the grid) was significantly reduced in the shield1-treated mice compared to controls (Fig. 4c) revealing reduced acquisition of context memory as a consequence of pharmacogenetic DG granule cell inhibition.

In order to exclude unspecific effects of shield1 and DD-Kir2.1 expression on the conditioning process itself, we assessed freezing before tone-shock pairing within the shock context (i.e., 3min pre-conditioning period), but we did not observe any treatment effects on freezing levels (freezing [% of total time; mean ± S.E.M.]: DD-Kir2.1-/shield1- = 1.00 ± 0.32; DD-Kir2.1 + /shield1- = 0.82 ± 0.22; DD-Kir2.1 + /shield1 +  = 0.76 ± 0.30; ANOVA: F_2,26_ = 0.19; p = 0.82).

In separate experiments, shield1 treatment (bath application for 2–3 h; 500 nM) of AAV-DD-Kir2.1 transduced DG granule cells significantly increased the RMP (no shield1: 74.5 ± 1.9 mV, shield1: 80.7 ± 1.1 [mean ± S.E.M.], t-test: t_10_ = 2.51; p = 0.03) and reduced *R*_in_ (no shield1: 67.40 ± 13.6 MΩ, shield1: 37.6 ± 9.6 MΩ [mean ± S.E.M.], t-test: t_8_ = 1.8; p = 0.06; n = 6 cells per group) compared to AAV-DD-Kir2.1 DG cells without ligand application as determined by intracellular recordings in acute brain slices. Of note, similar results were obtained by constitutive lentiviral overexpression of Kir2.1 in hippocampal CA1 region^15^.

## Discussion

In the past years, the technical abilities to manipulate neuronal substrates controlling brain function and behavior in the living animal have increased tremendously. Pharmaco- and optogenetic techniques with chimeric designer receptors or exogenous channels provide the unique opportunity to bi-directionally control neuron excitability and activity on a cellular and circuit level (for review ref. 1 and 3). Other neurotechniques can be applied to interrogate proteins of interest in a targeted fashion to study their neurobiological effects. For instance, conditional reverse genetics using the Cre-Lox technology is very useful to inactivate genes in mice, but this approach is not reversible. In order to achieve temporal control, transcription of the gene of interest can be regulated using a small-molecule-dependent promoter (e.g., tetracycline-regulated transactivator)^16^. Posttranscriptionally, protein levels can be perturbed by targeting mRNA by means of RNA interference^17^.

Targeting precursor DNA or mRNA provides high specificity, but not reversibility and rapid kinetics. Therefore, experimental strategies have been developed to target protein of interests directly (for review ref. 18 and 19). In an elegant fashion, one of them uses small-molecule induced protein stabilization. Here, genetic fusion of protein of interest with a mutant of the human FKBP12 (DD), which is engineered to be metabolically unstable, produces a chimeric protein. The instability of DD conferred to the fused partner protein induces its degradation by the proteasome, which can only be inhibited by adding the cell-permeable, synthetic ligand shield1^5^. Several *in vitro* studies could demonstrate shield1 - DD system-related stability in a wide range of cytoplasmic, nuclear or membrane proteins, e.g. kinases, cell cycle regulatory proteins^5^, proteins essential in herpesvirus infection^20^, MHC class I antigens^21^ or channels (TRPV5)^22^. *In vivo*, this technique has also been applied with success in parasites^23^ and in mice to control metabolic proteins^24^ or immunmodulatory cytokines with effects on tumor burden reduction^6^. Like the FKPB-derived system, unstable *Escherichia coli* DHFR mutants and their stabilizing small molecule, trimethoprim, have been developed and recently used to stabilize the expression of Cre recombinase in the mouse brain^7^. The ligand trimethoprim, however, is not biological inert, but has a strong, clinically used antimicrobial potency. As there is growing evidence for an effect of antimicrobial agents on gastrointestinal microbiota interacting with brain function^25^, such a source of putative experimental bias needs to be considered in neurobehavioral studies^26^.

**Figure 5:**
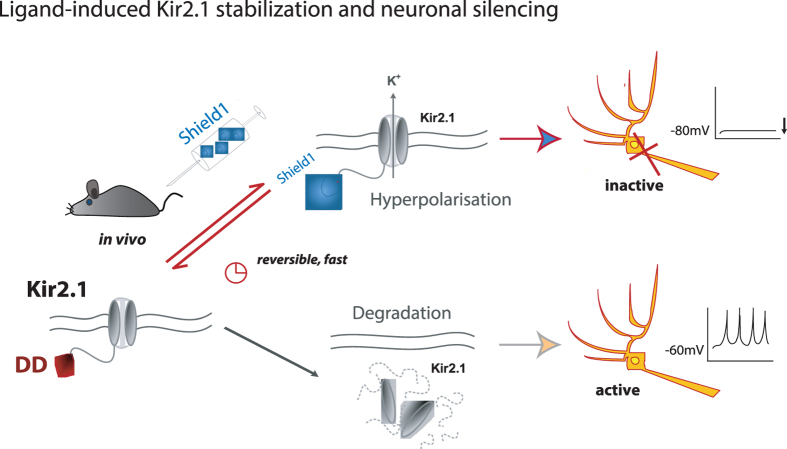
Schematic of the principle of shield 1-induced Kir2.1 stabilization in neurons and neuronal silencing in living animals.

Here, we use the shield1-DD system to control the potassium channel subtype Kir2.1 in neurons, and develop a novel pharmacogenetic tool for reversible and remote inhibition of neuronal cells and circuits *in vitro* and in the living animal (Fig. 5). Encoded by the KCNJ2 gene, Kir2.1 is an inwardly rectifying potassium channel that stabilizes the resting membrane potential and modulates excitability in neurons, but it is also expressed in cardiac myocytes and skeleton muscles^8^. In the central nervous system, Kir2.1 is expressed diffusely in the whole brain, but restricted to neuronal somata and dendrites on the cellular level^27^,^28^. Ectopic expression of the Kir channels has repeatedly been used to inhibit excitability of neurons or neuronal networks and to study its effects on circuit function^10^,^11^,^29^. By endowing Kir2.1 with ligand-mediated stability, we have provided the ability for spatial and temporal control. Shield1-induced Kir2.1 stabilization works in the cell culture as shown in electrophysiological recordings. We also used an adenoviral vector to transfer the construct into different brain areas. Upon small molecule administration, the fusion protein DD-Kir2.1 was expressed and neurons displayed a hyperpolarized RMP, a decreased *R*_in_ and a higher rheobase. These effects were observed in different neuronal cell types. The ligand shield1 itself, previously described to be biological inert, did not affect neurophysiological characteristics in native neurons.

We have evaluated DD-Kir2.1’s efficacy as electrical silencer of target neurons also in the behaving animal. We observed robust behavioral responses in the AMP-induced rotation test that is highly sensitive to perturbations of neuronal activity in the striatum^2^. Importantly, the systemic injection of shield1 evoked unilateral rotations only at an early time point of administration. At later time points, we did not observe a phenotype indicative of suppression of neuronal excitability. Likewise, DD-Kir2.1 was significantly upregulated at the site of expression only 8h after shield1 treatment and is in accordance to previously published findings on shield1-mediated secretion of cytokines in a cancer mouse model^6^. With respect to pharmakokinetics, the small molecule could only be detected directly in the mouse brain at this time point. Here, we did not study earlier time points for behavioral analysis or pharmakinetics because maximal *in vivo* effects were reported to occur between 8 and 12 h after ligand administration^6^. Due to technical and biological limitations, electrophysiological recordings after an 8 to 12 h incubation period of acute slices with shield1 was not feasible. As compared to our slice electrophysiology data, effects on DD-YFP stabilization in NIH3T3 cells were also observed already after 4h as earliest time point assessed^5^.

Nevertheless, our data together with the results described by Banaszynski *et al.* (refs 5 and 6) suggest a relatively slow onset kinetics. However, the decay of shield1 effects occurs within 24h after a single application *in vivo* and likely reflects the combined influence of ligand clearance and turnover of the fusion protein. The slow onset kinetics, however, is in complete contrast to ultra-rapid acting optogenetic methods for neuronal silencing. This lack of precise temporal control is a limitation when it comes to dissect brain circuits involved in certain behaviors, e.g., social behaviors. Nevertheless, the kinetics and the persistence of shield1 effects over a few hours may make it especially attractive for long-term modulations of neural circuits. As an example, neuroscience research on chronic neurobiological processes involved in epilepsy, depression or acquisition processes during memory consolidation can be mentioned. In the present study, we are able to provide one example of an animal study, where we interfere with hippocampus-dependent fear memory formation in the living mouse.

Sustained suppression of neuronal firing *in vitro* and *in vivo* was also reported for a photo-inducible mutant version of Kir2.1 4. This method, however, lacks reversibility, which limits its use in behavioral neurosciences. Of note, another chemogenetic tool offers reversible neuron inhibition, and is frequently applied in neuroscience research. Activation of the inhibitory DREADD (designer receptors exclusively activated by designer drugs) hM4Di by their inert ligand clozapine-N-oxide stimulates Gi coupled GPCRs that further activate inwardly rectifying potassium channels^30^. In contrast to genetic Kir2.1 manipulations and its specific effects on the membrane potential, activation of hM4Di also leads to other downstream signalling events, including the activation of the ERK/MAPK pathway, or it directly interferes with neurotransmitter release^3^. An additional confound may arise when DREADDs, but also the FKPB system, are highly overexpressed as they exceed the physiological levels of endogenous GPCRs with incorrect targeting^3^. With respect to the FKPB system, an excessive DD-protein of interest copy number may exceed the degradation capacity of the proteasome of the individual cell as reported by Sando *et a*l. (ref. 7). In our study, we observed in an *in vitro* experiment (Western blot; see Fig. 1c) and in the slice electrophysiology basal expression and activity of DD-Kir2.1 (e.g., RMP; see Fig. 2c) without shield1 treatment, which indicates small leakage or incomplete proteasomal degradation. However, this basal expression did not significantly influence behaviour as observed in the fear conditioning experiment.

In summary, we used an existing tool, shield1-DD system, and present its applicability for *in vivo* brain research. By combining it with a physiological hyperpolarizing mechanism it also presents a novel pharmacogenetic tool that allows remote, reversible and specific inhibition of neuronal cells. An important advantage of using the shield1-DD system over existing chemogenetic techniques is that it modulates a channel whose intrinsic function is to regulate neuron excitability. The ligand is biologically inert, has no toxic side effects, is commercially available and possesses good pharmacokinetic characteristics including penetration of the blood-brain barrier. Finally, the method described in the present study may not only be utilized for studying biological processes in the brain, but also for research of physiological processes and diseases that directly involve Kir2.1 like the Andersen-Tawil syndrome, a cardiac long QT syndrome^31^. By demonstrating its *in vivo* potentials, this system may be of interest for many other experimental applications and protein of interests, e.g., intracellular kinases (CaMKII or PKMζ) in the field of neurobehavioral research.

## Methods

### Animals

Experiments were performed with male *C57BL/6NCrl* (B6) inbred mice purchased from Charles River Germany at an age of 6–8 weeks. All animals were kept under standardized single housing conditions (i.e., Makrolon type II cages with wood shavings, inverse 12 h light/dark cycle with lights off at 0900 hours, at 22 ± 2 °C room temperature and 55 ± 5% humidity). Water and food were provided *ad libitum*. Animals were allowed to habituate to the local housing conditions for at least 2 weeks before starting the experiments.

All experimental procedures were performed in accordance with the guidelines for the care and use of laboratory animals set by the European Community Council and approved by the local animal welfare authority (Regierung von Oberbayern).

### *In vitro* and viral vector constructions

#### Cloning and production of *in vitro* vectors

Cloning and mutagenesis was performed by standard techniques. Coding sequences of the human Kir2.1 (ImaGenes, Berlin, Germany) and the DD-ZsGreen1-Tag (Clontech Laboratories, Saint-Germain-en-Laye, France) were subcloned in a first step in a pcDNA3.1-plasmid (Invitrogen) to get the DD-ZsGreen1-Kir2.1 in pcDNA3.1-plasmid. We used as primers pDDfor_glu (GCGATCGGAT-CCGCCGCCACCATGGGAGTGCAGGTGG-AAACCATC), pDDrev_glu (CCCAGAGAATT-CGGGCAAGGCGGAGCCGGA), hukirfor_glu (GCGATCGAATTCGGCAG-TGTGCGAACCAAC), hukirrev_glu (CCCAGACTCGAGTCATATCTCCGACTCT-CGCCGTAA). For *in vitro* electrophysiological experiments we constructed a second *in vitro* vector carrying just DD-Kir2.1 within a pIRES-eGFP-plasmid (Clontech Laboratories). Therefore, in a first step, we cut out the ZsGreen1 from the DD-ZsGreen1-Kir2.1 in pcDNA3.1-vector by using DD-rev (CCCAGAGAATTCTTCCGGTTTTAGAAGCTC). Second, we subcloned DD-Kir2.1 in the pIRES-GFP-plasmid by using DDkircutfor (GCGATCGCTAGCGCCGCCACCATGGGAGTGCAGGTGGAAACCATC) and DDkircutrev (CCCAGAGTCGACTCATATCTCCGACTCTCGCCGTAA).

#### Cloning and production of rAAV vectors

Cloning and mutagenesis was performed by standard techniques. All sequence manipulations were confirmed by sequencing. The 1.3kb CamKIIalpha promoter^12^ was PCR cloned into the AAV2.1-msc-WPRE plasmid^32^ to obtain pAAV2-1-CamKII-msc-WPRE. To construct pAAV-CamKII-DD-Kir2.1-WPRE, we inserted the PCR amplified human DD-Kir2.1 DNA fragment (1.6 kb BsrgI-EcoRV fragment from the RosaTV plasmid, see vector for *in vitro* experiments) (DD-Kir2.1-BsrgI-for: 5′-GCGATCTGTACAGCCGCCACCATGGGAGTGCAGGT -3′ and DD-Kir2.1-EcoRV-rev: 5′-CCCAGAGATATCTATCTCCGACTCTCG -3′) into pAAV2.1-CamKII-msc-WPRE, respectively. Subsequently, a PCR product containing the PTV1.2A sequence^33^ and eGFP coding sequence was cloned blund end into the EcoRV site between DD-Kir2.1 and the WPRE sequence of pAAV-CamKII-DD-Kir2.1-WPRE to generate pAAV-CamKII-DD-Kir2.1-2A-GFP-WPRE. A schematic representation of the AAV2.1-CamKII-DD-Kir2.1-2A-GFP-WPRE vector is given in Fig. 2A.

Single-strand recombinant AAV8YF-pseudotyped AAV were produced by triple calcium phosphate transfection of 293T cells with pAdDeltaF6^34^, and pAAV2/8Y33F^35^ and pAAV2.1-CamKII-DD-Kir2.1-2A-GFP-WPRE plasmid followed by iodixanol-gradient purification as described previously^32^,^36^. Physical titers (in genome copies/ml) were determined by quantitative PCR of WPRE using a LightCycler 480 (Roche Applied Science, Mannheim, Germany).

### Stereotactic brain surgery and virus injection

Mice were anesthetized with isoflurane (Forene^®^, Abbott, Germany) and placed in a stereotaxic apparatus (TSE Systems, Germany) with adapted components to allow mouse inhalation anesthesia. After exposure of the skull, holes were drilled for virus infusions according to the following coordinates adapted from a stereotactic brain atlas^37^: dorsolateral thalamus (unilateral injections): -1.6 mm posterior to bregma, + 1.2 mm lateral from midline and +4.0 mm below the surface of the skull; striatum (unilateral injections): +0.0 mm posterior to bregma, +2.0 mm lateral from midline and +4.2 mm (injection cannula retracted to +2.5 mm during the injection to cover the dorso-ventral extension of the area) below the surface of the skull; dorsal hippocampus (bilateral injections): −1.6 mm posterior to bregma, ±1.0 mm lateral from midline and +2.0 mm below the surface of the skull.

The AAV was used at a titer of 1,96 × 10E10 vg/μl. We injected 1μl of AAV-containing solution per side into the thalamus and hippocampus and 1.5 μl into the striatum at infusion rates of 70 nl/min using a microinfusion pump. After infusion, the cannula was left in the brain for another 10 min to allow virus diffusion before removal.

Mice received analgesic treatment before surgery (meloxicam 0.5 mg/kg, s.c.; Metacam^®^, Boehringer Ingelheim, Germany) and 3 days afterwards by drinking water at the same dose. Animals were allowed to recover from surgery for 3–4 weeks before starting the experiments.

### Electrophysiology

#### Recordings in acute brain slices

Mice were anesthetized with isoflurane and decapitated, the brains were quickly transferred in ice-cold carbogenated (95% O_2_/ 5% CO_2_) artificial cerebrospinal fluid (aCSF) with low amount of Ca^2+^ and a high amount of Mg^2+^ containing in (mM) 125 NaCl, 2.5 KCl, 1.25 NaH_2_PO_4_, 25 D-glucose, 25 NaHCO_3_,6 MgCl_2_, 0.5 CaCl_2_.

Transversal thalamic and coronal hippocampal slices (350 μm) were performed using a vibroslicer (HM 650 V, Microm International, Walldorf, Germany). Slices were transferred to aCSF containing (in mM) 125 NaCl, 2.5 KCl, 1.25 NaH_2_PO_4_, 25 D-glucose, 25 NaHCO_3_, 1 MgSO_4_, 2 CaCl_2_ and allowed to recover for at least 1 h at 34 °C before being transferred to the recording chamber where they were continuously superfused with aCSF at a rate of 2 ml/min. Saturation with a mixture of 95% O_2_/5% CO_2_ (carbogen gas) led to a pH of 7.4. All experiments were performed at room temperature (22–24 °C).

Whole cell patch-clamp recordings in acute slices from mice (aged 8-11 weeks [3-4 weeks after virus injection]) were obtained from pyramidal neurons in the dorsolateral thalamus or from dentate gyrus granule cells by using infrared guided videomicroscopy. The recording pipettes (resistance between 4 and 6 MΩ) were filled with (in mM) 130 K-D-gluconate, 5 NaCl, 0.5 EGTA, 2 Mg-ATP, 10 HEPES, 5 D-glucose. Recording was done at room temperature.

Identification of transfected excitatory neurons was able by detecting the GFP expressed by the vector. Excitation wavelength was 488 nm (XC Polychrome V; T.I.L.L. Photonics), GFP-signal was registered by a High Speed CCD Kamera (Retiga-2000RV, Qimaging) and recorded and displayed by the program TillVision (T.I.L.L. Photonics). Currents and potentials were recorded by a discontinuous voltage clamp amplifier (SEC 10, NPI electronics, Tamm, Germany) in the voltage-clamp (VC) mode (switching frequencies 40–43 kHz, 25% duty cycle) and bridge mode, respectively. Cells were incubated with extracellular shield1 (500 nM) for at least 2 h. We measured the resting membrane potential (RMP) in neurons of acute brain slices in bridge mode. Afterwards, we performed current injections in the current clamp (CC) mode with 10 pA incremental steps starting at −90 pA up to 100 pA to define a current-voltage (IV) relationship. In another set of experiments, depolarization steps (10pA; CC mode) up to 400 pA were used to trigger action potential firing and to determine the rheobase. The holding potential was set at −70 mV.

#### Recording in HEK293T cells

24 h after transfection with the DD-Kir2.1 in pIRES-EGFP plasmid, cells were trypsinised and seeded on 12 mm cover slides covered with Polylysine A. 24 h later, patch clamp experiments were performed. These experiments were performed at room temperature (22–24 °C). In the recording chamber the cells were superfused continuously with an extracellular solution containing (in mM) 135 NaCl, 5.4 KCl, 1.8 CaCl_2_, 1 MgCl_2_, 10 D-glucose, 5 HEPES. The solution was saturated with carbogen gas (95% O_2_/ 5% CO_2_) and had a pH of 7.4. Pipettes were filled with internal solution containing (in mM) 130 KCl, 5 NaCl, 2 MgCl_2_, 5 EGTA, 0.2 MgATP, 5 HEPES. Shield1 was applied to the extracellular solution 4–8h before recordings. Transfected cells were identified by the EGFP-signal of the vector using an excitation wavelength of 488 nm (XC Polychrome V; T.I.L.L. Photonics), recorded with a High Speed CCD Kamera (Retiga-2000RV, Qimaging) and displayed by the program TillVision (T.I.L.L. Photonics). In HEK293T cells, we recorded only the resting membrane potential.

### Western blot analyses

Animals were stereotactically injected with AAV-DD-Kir2.1 into the right striatum and were treated either with shield1 (10 mg/kg; i.p.) or saline (i.p.). At different time points after drug injections (8 h, 24 h or 48 h), mice were sacrificed, brain extracted and frozen. For striatum dissection, brains were cut with a cryostat up to the appearance of the striatum area. Specimens of both sides were isolated using cylindrical punchers (Fine Science Tools, Heidelberg, Germany). Thereafter, striatal specimens were frozen in liquid nitrogen. The frozen tissue was than powdered using ultrasound and pestle after mixing with 2% SDS/50mM Tris, pH 7,4 (100 μl/10 mg tissue) and heated for 15 min at 95 °C. The solution was centrifuged at 20.000 g for 5 min. The supernatant containing tissue proteins was frozen at −80 °C and used for immunoblotting (10 mg tissue).

Immunoblotting was done as described previously^14^. Protein samples for Western blotting were separated on an 8% SDS-polyacrylamide gel and transferred to a PVDF membrane. For protein detection the following antibodies were used: a mouse monoclonal anti-DD antibody (1:500; Clonetech Laboratories, Saint-Germain-en-Laye, France) and a rabbit polyclonal anti-p42/44-MAPK antibody (1:1000; Cell Signaling, Leiden, Netherlands).

### Image acquisition

After the experiments, animals were sacrificed and viral spread was assessed by auto-fluorescence using fluorescent microscopy (Zeiss Axioplan 2 microscope) using 10×, 40× and 60× oil-immersion objectives. Digital images were processed using Zeiss Axioplan software or NIH-Image J software package. Only data of mice with an orthotop viral expression were included in further analyses.

### Drugs

For systemic drug administration, mice were treated intraperitoneally (i.p.) with 10 mg/kg shield1 (Clontech Laboratories, Saint-Germain-en-Laye, France; Cheminpharma, Branford, U.S.), 2.5 mg/kg d-amphetamine sulfate (Sigma Aldrich, Germany) dissolved in sterile saline with a volume of 0.1 ml/10 g.

### Behavioral analyses

Animals were transferred from the vivarium to the behavioral laboratory 1–2 days before an experiment, where they were kept and tested under identical laboratory conditions as in the housing facility. All experiments were performed during the activity period of the mice (i.e., dark phase of the light cycle) between 0930 and 1700. Experiments and behavioral analyses were performed by experienced experimenters unaware of the experimental groups.

#### Amphetamine-induced rotation test

Testing was performed in an open field (OF) (26 × 26× 38 cm^3^; TruScan, Coulbourn Instruments, Allentown, PA) made of a white floor and transparent Plexiglas walls and equipped with two infrared sensor rings to measure horizontal and vertical movements of an animal. Photo beams were connected via interface to a PC running a TruScan software (V.99; Coulbourn Instruments). The test arena was illuminated with 20 lux. Boxes and sensor rings were surrounded by an additional box made of opaque Plexiglas walls without roof and floor.

For testing amphetamine (AMP)-induced rotations, animals received either vehicle (saline, i.p.) or shield1 (10 mg/kg; i.p.) 8 h before. Then, they were placed into the margin zone of the arena and allowed to move freely in the arena for 10min (baseline movements). Immediately afterwards, all mice received AMP (2.5 mg/kg, i.p.; dose according to Lerchner *et al.* [ref. 2]) and placed back to the OF arena for additional 40 min. Their movements were automatically recorded during the whole testing period. After the trial, animals were returned to their home cages and the boxes were thoroughly cleaned with water containing liquid soap and rinsed with clear water. Additional trials were done 24 h and 48h after the shield1/saline-injections without *de novo* ligand application. Using the TruScan software package, general locomotion was assessed by measuring the total distance moved, and the total number of ipsilateral rotations was scored manually as an index of AMP-induced stereotype behavior.

#### Fear conditioning

The experimental procedures and the equipment used for fear conditioning were previously described in detail^14^. Briefly, tone-shock pairing was performed in a mouse-conditioning chamber (MED Associates, St. Albans, VT) with a cubic-like shape and a metal grid floor for shock application. Tones were generated by an audio stimulus generator (MED Associates) and applied by top-mounted speakers. Contexts for conditioning and testing were located in soundproof boxes (MED Associates). Exact timing and application of foot shocks, sounds and light were executed and controlled electronically by an interface-connected PC running MED-PC software for Windows (MED Associates).

In detail, 8 h before fear conditioning, animals received either vehicle (saline, i.p.) or shield1 (10 mg/kg; i.p.). Then, mice were placed into the conditioning chamber and the house light (10 lux) was switched on. After a habituation phase of 180 sec, a sine wave tone (80 dB, 9 kHz) was presented for 20 sec that co-terminated with a 2-sec, scrambled electric foot shock (unconditioned stimulus [US]) of 1.5 mA intensity. Animals remained in the shock context for additional 60 sec before they were returned to their home cages. To assess auditory fear memory 24 h later, a different test context was used consisting of a Plexiglas cylinder. Instead of a grid, the floor was made of plane PVC and covered with wood shavings. After 180 sec, animals were exposed to a continuous 3-min tone (80 dB, 9 kHz), and returned to their home cages additional 60 sec later. To test for contextual fear memory, the original shock context was used, and in terms of assessment of only a context feature a slightly different test box consisting of a hexagonal shaped prism made of non-transparent Plexiglas with a metal grid floor as a dominant reminder of the shock-context was used. In these tests, done 2–4 h after tone memory testing, mice were placed in these contexts for 3 min without receiving additional foot shocks. After each test, all three contexts were cleaned thoroughly with differently smelling detergents, and bedding was changed. Behavior during the conditioning and testing sessions was recorded on DVD for further off-line analysis. As a measure of fear, we assessed freezing behavior defined as the absence of all movements except for respiration and the animal’s head remaining in a horizontal position. Freezing was scored by a trained observer by means of customized freeware software (EVENTLOG by Robert Henderson, 1986).

### Analysis of shield1 by mass spectrometry

C57BL/6 mice received shield1 (10mg/kg) i.p. and were killed with an overdose of isoflurane at different time points afterwards. Before brain extraction, blood was taken by heart puncture, and animals were perfused with PBS for 2 min. Brains were weighed and then homogenized in a fivefold volume PBS containing a protease inhibitor cocktail (Roche, Germany) using a Dispomix Drive (Medic Tools, Switzerland).

Serum and brain homogenates were analysed using the combined high-performance liquid chromatography/mass spectrometry (HPLC/MS-MS) technique. Analysis was performed using an Agilent 1100 Series (Agilent, Germany) liquid chromatograph which was interfaced to the ESI source of an Applied Biosystems API 4000 (ABSciex, Germany) triple quadrupole mass spectrometer. All samples were prepared using Ostro protein precipitation and phospholipid removal plates (Waters, Germany).

Deuterated clomipramine (Clomi-D3) was used as internal standard. Chromatography was accomplished using an gradient elution in a Accucore RP-MS 2,6 μm column (2.1 × 50 mm, Thermo Scientific, Germany) at a flow rate of 0.3 ml/min and 30 °C. The composition of eluent A was methanol with 10 mM ammonium formate with 0,1% formic acid and water with 10 mM ammonium formate with 0,1% formic acid as eluent B. The gradient was 0–0,5 min 55% A, 0.5–2 min 55–90% A, 2–3 min held at 90% A, 3–3.5 min 90–55% A and 3.5–8min 55% A. The total run time was 8 min and the injection volume was 5 μl.

The retention time for shield1 was 4,1 min and 4,5 min for Clomi-D3. The ion source was operated in the positive mode at 500 °C, and multiple reaction monitoring (MRM) collision-induced dissociation (CID) were performed using nitrogen gas as the collision gas. The collision energy was set to 43 V for shield1 and 27 V for Clomi-D3. The transitions monitored during analysis were *m/z* 762 → 397 for shield1 and *m/z* 318 → 89 for Clomi-D3. The detection limit for shield1 in the serum was 10 ng/ml and 1.5 ng/g wet weight in brain tissue.

### Statistical analyses

Data are presented as mean ± S.E.M. Data were analyzed by two-group comparisons using unpaired Student’s t-tests, and one-way analyses of variance (ANOVA) in case of multiple group comparisons or ANOVAs with repeated measures when repeated within-subjected measurements performed. Posthoc analyses were done by the Newman-Keuls test. Analyses were performed using GraphPad Prism 5.0 (GraphPad, CA) and SPSS 18.0 (SPSS Inc, IL). Statistical significance was accepted if p ≤ 0.05.

## Additional Information

**How to cite this article**: Auffenberg, E. *et al.* Remote and reversible inhibition of neurons and circuits by small molecule induced potassium channel stabilization. *Sci. Rep.* **5**, 19293; doi: 10.1038/srep19293 (2015).
